# 5A Zirconium Dioxide Ammonia Microsensor Integrated with a Readout Circuit Manufactured Using the 0.18 μm CMOS Process

**DOI:** 10.3390/s130303664

**Published:** 2013-03-15

**Authors:** Guan-Ming Lin, Ching-Liang Dai, Ming-Zhi Yang

**Affiliations:** Department of Mechanical Engineering, National Chung Hsing University, Taichung 402, Taiwan; E-Mails: hunter741213@yahoo.com.tw (G.-M.L.); d099061005@mail.nchu.edu.tw (M.-Z.Y.)

**Keywords:** integrtaed ammonia microsensor, zirconium dioxide, readout circuit, CMOS

## Abstract

The study presents an ammonia microsensor integrated with a readout circuit on-a-chip fabricated using the commercial 0.18 μm complementary metal oxide semiconductor (CMOS) process. The integrated sensor chip consists of a heater, an ammonia sensor and a readout circuit. The ammonia sensor is constructed by a sensitive film and the interdigitated electrodes. The sensitive film is zirconium dioxide that is coated on the interdigitated electrodes. The heater is used to provide a working temperature to the sensitive film. A post-process is employed to remove the sacrificial layer and to coat zirconium dioxide on the sensor. When the sensitive film adsorbs or desorbs ammonia gas, the sensor produces a change in resistance. The readout circuit converts the resistance variation of the sensor into the output voltage. The experiments show that the integrated ammonia sensor has a sensitivity of 4.1 mV/ppm.

## 1. Introduction

Ammonia sensors are already widely applied in industrial and environmental monitoring. Various microsensors have been developed using microelectromechanical system (MEMS) technology, and the advantages of their microsensors are small size, high performance low cost and easy mass-production [1]. Several ammonia microsensors have been fabricated using MEMS technology. For instance, Lee *et al.* [2] utilized bulk micromachining to make an ammonia microsensor. The sensitive film of the sensor was polyaniline. The sensor was composed of a SU-8 adhesion layer, a sensitive film and interdigitated Pt electrodes. The ammonia sensor had a sensitivity of about 40% at 50 ppm ammonia. Yoon *et al.* [3] fabricated an ammonia microsensor using MEMS technology. The sensitive material of the sensor was palladium doped tungsten trioxide synthesized by the sol-gel method, and the material was coated on the micro platform and annealed at 400 °C. Carquigny *et al.* [4] developed a gas microsensor for detecting ammonia gas. The polypyrrole was adopted as a sensitive material that was deposited by electrochemical process. An ammonia sensor, proposed by Patois *et al.* [5], was manufactured using silicon microtechnology, and the sensitive film of polypyrrole was electrochemically deposited on platinum microelectrodes. Triantafyllopoulou *et al.* [6] used porous silicon technology to produce the ammonia sensors. Two different metal-modified nanostructured sensitive materials, SnO_2_/Pd and WO_3_/Cr, were deposited on the active area of the micro-hotplates, and the SnO_2_/Pd sensor was more sensitive to ammonia. Llobet *et al.* [7] employed screen-printing technique to fabricate the gas sensors with a polysilicon heater for detecting ammonia gas. The sensitive material of nanopowder tin oxide was deposited on the platinum electrodes on silicon substrates. The ammonia sensors, presented by Lee *et al.* [2], Yoon *et al.* [3], Carquigny *et al.* [4], Patois *et al.* [5], Triantafyllopoulou *et al.* [6] and Llobet *et al.* [7], were not integrated with circuitry on-a-chip. The advantages of microsensors integrated with circuitry on-a-chip are low package cost and high performance [1]. In this work, we fabricate an ammonia sensor integrated with readout circuit on chip. Comparison to capacitive ammonia sensors, the advantages of resistive ammonia sensors include that the output signal has a high linearity and has no parasitic capacitance problem.

The CMOS-MEMS technique uses the commercial CMOS process to develop MEMS devices [8,9]. Microdevices fabricated by this technique can be integrated with circuits as a system on a chip (SOC). In this study, an ammonia sensor with a readout circuit on chip is developed utilizing the CMOS-MEMS technique. Zirconium dioxide is adopted as the sensitive material of the sensor since it has a high selectivity and a high sensitivity for ammonia gas [10,11]. The sensor needs a post-process to deposit the sensitive material. The post-process consists of etching the sacrificial oxide layer and depositing the sensitive film.

## 2. Structure of the Ammonia Sensor

**Figure 1.**
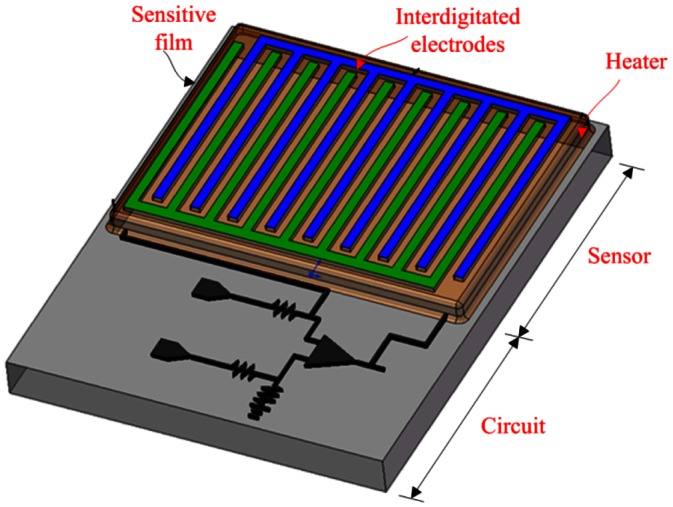
Schematic of the integrated ammonia sensor.

The integrated microsensor chip is composed of an ammonia sensor, a heater and a readout circuit. Figure 1 illustrates a schematic of the integrated ammonia sensor chip. The area of the integrated sensor chip is 1.5 mm^2^. The ammonia sensor is made of a sensitive film and interdigitated electrodes. The sensitive film material is zirconium dioxide coated on the interdigitated electrodes. The interdigitated electrodes are 600 μm long, 15 μm wide and 6 μm thick, and the gap between the electrodes is 10 μm. The heater located under the interdigitated electrodes is employed to provide a working temperature to the sensitive film, and material of the heater is polysilicon. The reaction mechanism of zirconium dioxide adsorbing ammonia gas is given by [11],

(1) $$O_{2\left(\text{air}\right)}+4e^{-}\to 2O^{2-}$$

and:

(2) $$Z_{r}O_{2}+5NH_{3}+4O^{2-}\to Z_{r}{\left(NH_{4}OH\right)}_{3}+2NO_{2}+8e^{-}$$

where *O^2−^* denotes the oxygen ion on the surface of zirconium dioxide film and *e^−^* is a conduction electron. According to Equations (1) and (2), when the sensitive film of zirconium dioxide adsorbs ammonia gas, the mobility of the film increases because the electrons in the film increase, leading to the resistance of the film decreases. Therefore, the ammonia sensor is a resistive type. The resistance of the ammonia sensor generates a change when the sensitive film adsorbs or desorbs ammonia gas. As shown in Figure 1, the integrated sensor contains a readout circuit that it is utilized to convert the resistance variation of the ammonia sensor into the output voltage.

**Figure 2.**
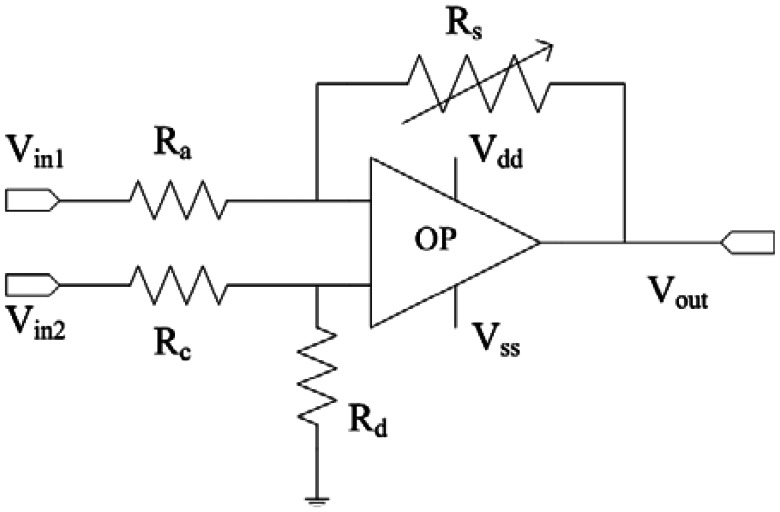
The readout circuit for the ammonia sensor.

**Figure 3.**
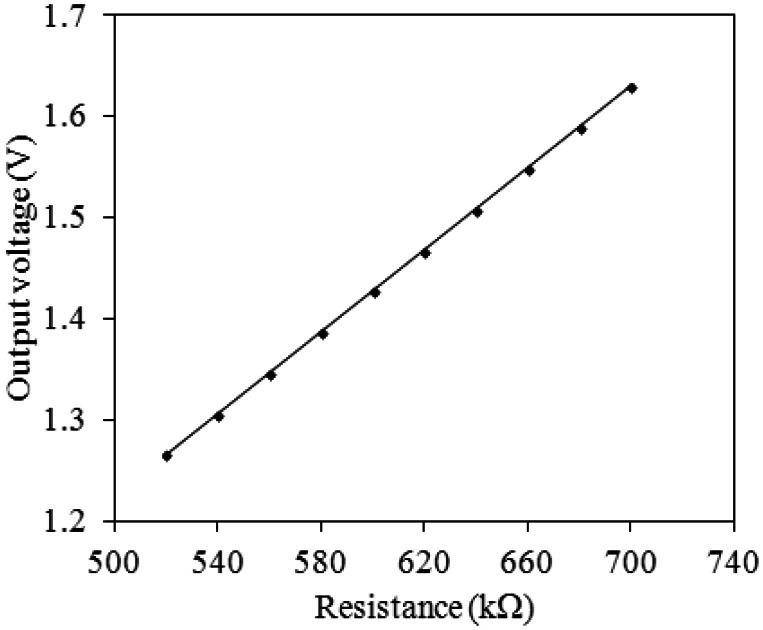
Simulated readout of the output voltage for the readout circuit.

**Figure 4.**
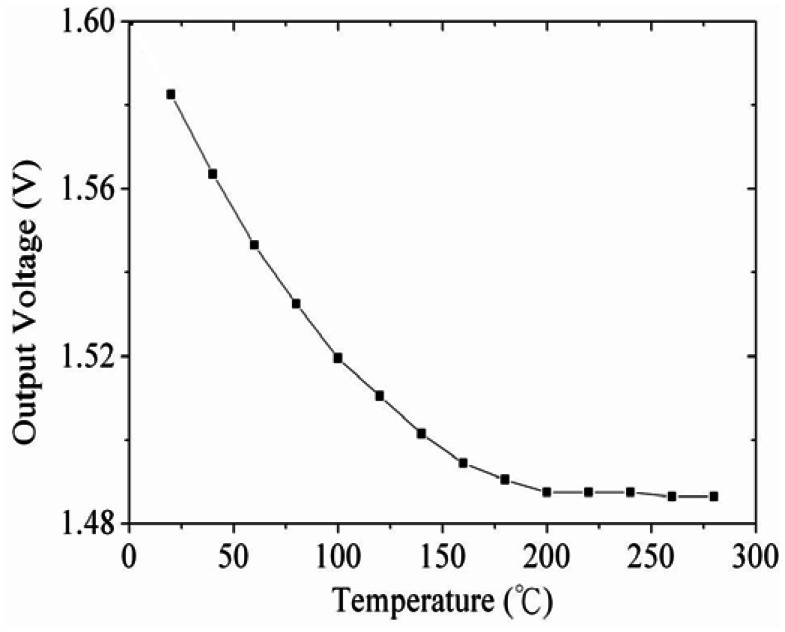
Relation between the output voltage and temperature for the circuit.

Figure 2 shows the readout circuit for the integrated ammonia sensor [12], where V_out_ represents the output voltage of the circuit; V_in1_ and V_in2_ are the input voltages of the circuit; V_dd_ is the bias voltage of amplifier; V_ss_ is the ground of amplifier; R_s_ is the resistance of the ammonia sensor; R_a_, R_c_, and R_d_ are the resistances of the circuit. The professional software, HSPICE, is employed to simulate the characteristics of the readout circuit. Figure 3 reveals the simulated results of the readout circuit for the ammonia sensor. In this simulation, the resistances R_a_ = 3 kΩ, R_c_ = 3 kΩ and R_d_ = 3 kΩ are given, and the input voltages V_in1_ and V_in2_ are 0.6 V and 0.9 V, respectively. The bias voltage of 3.3 V is adopted. The resistance of the ammonia sensor changes from 700 to 520 kΩ. The simulated results show that the output voltage of the readout circuit decreases from 1.58 to 1.31 V as the resistance of the sensor changes from 680 kΩ to 540 kΩ. In order to characterize the influence of temperature to the circuit, the output voltage of the circuit is simulated at different temperatures. Figure 4 shows the relation between the output voltage and temperature for the readout circuit. In this investigation, the bias voltage V_dd_ is 3.3 V, and the input voltages V_in1_ and V_in2_ are 0.6 V and 0.9 V, respectively. The resistance of the ammonia sensor is 680 kΩ, and the resistance R_a_, R_c_, and R_d_ are 3 kΩ, 3 kΩ and 3 kΩ, respectively. The temperature varies from 25 to 280 °C. The results depict that the output voltage of the readout circuit decreases from 1.58 to 1.482 V as the temperature changes from 25 to 280 °C, in which the output voltage reduces 0.1 V.

## 3. Preparation of the Sensitive Film

The sensitive film of the ammonia sensor was zirconium dioxide prepared by the sol-gel method [13,14] as follows: zirconium *n*-propoxide (Zr (OC_3_H_7_)_4_, 70 wt%) precursor was dissolved in ethylene glycol (C_2_H_4_(OH)_2_) with stirring for 3 hours until the solution was mixed uniformly. The mixed solution was added into the NaOH precipitant with stirring for 1 hour. Then, the mixed solution with NaOH was mixed with aluminium nitrate and stirred for 1 hour, followed by a hydrothermal process for 12 hours at 90 °C. The slurry of zirconium dioxide was filtered, and then rinsed with deionized water. Finally, the zirconium dioxide was coated on the substrate with calcination at 100 °C for 1 hour.

**Figure 5.**
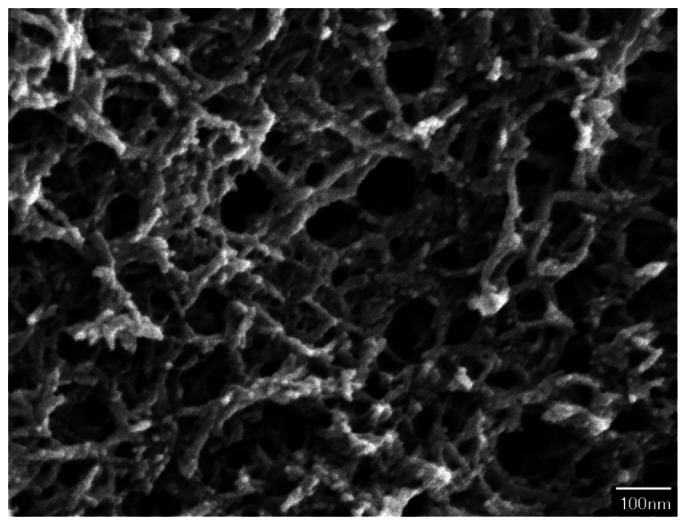
SEM image of the zirconium dioxide film.

The surface morphology of the zirconium dioxide film was measured by scanning electron microscopy (JEOL JSM-6700F). Figure 5 shows a scanning electron microscope (SEM) image of zirconium dioxide film. The film is a porous structure that has a large surface area and this helps to enhance its sensitivity. An energy dispersive spectrometer was used to measure the composition of the zirconium dioxide film. The results showed that the film contained 66.55 wt% zirconium and 33.45 wt% oxygen.

## 4. Fabrication of the Ammonia Sensor

**Figure 6.**
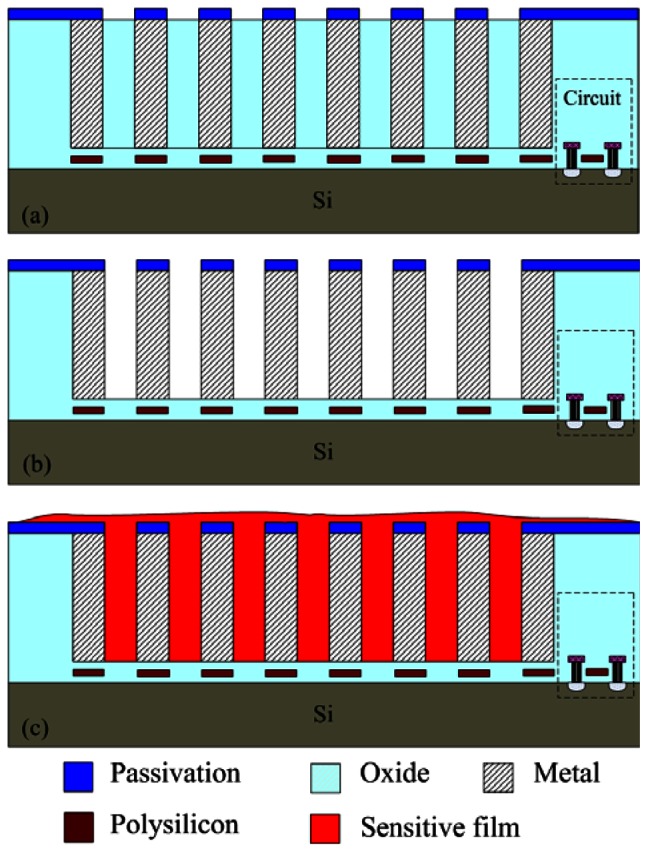
Fabrication process of the integrated ammonia sensor: (**a**) after the CMOS process, (**b**) etching the sacrificial layer, (**c**) the sensitive film coated.

The integrated ammonia sensor with a readout circuit was fabricated using the commercial 0.18 μm CMOS process of Taiwan Semiconductor Manufacturing Company (TSMC). Figure 6 illustrates the process flow of the integrated ammonia sensor. After completion of the CMOS process, a post-process was used to coat the sensitive film on the ammonia sensor. The post-process included two main steps: (1) the sacrificial layer was etched to expose the interdigitated electrodes; (2) the zirconium dioxide film was coated on the interdigitated electrodes.

**Figure 7.**
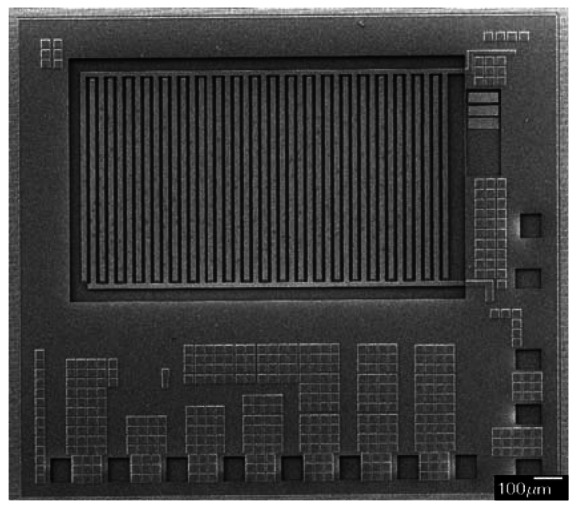
SEM image of the ammonia microsensor after the wet etching.

**Figure 8.**
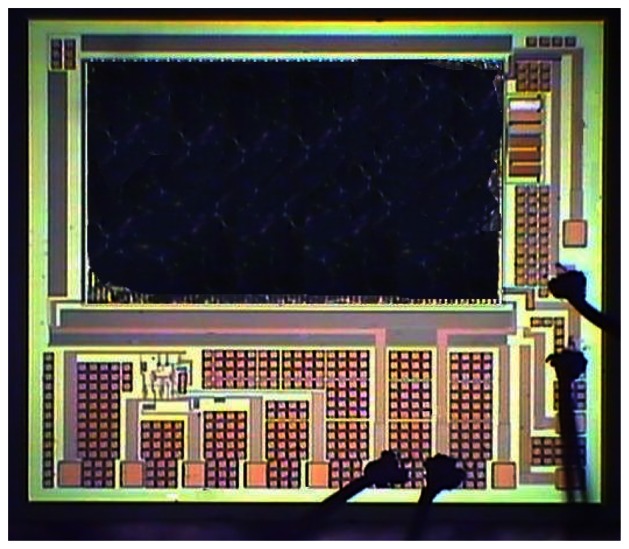
Optical image of the integrated ammonia sensor after the post-process.

Figure 6(a) shows the cross-section of the integrated ammonia sensor after the CMOS process. The sacrificial layer located between the interdigitated electrodes was silicon dioxide. As shown in Figure 6(b), a wet etching with BOE (buffer oxide etch) solution was utilized to etch the sacrificial layer of silicon dioxide, and to expose the interdigitated electrodes. Figure 7 shows a SEM image of the interdigitated electrodes after the wet etching. As shown in Figure 6(c), the zirconium dioxide was dropped on the interdigitated electrodes using a precision-control micro-dropper, and then the zirconium dioxide film was calcinated at 100 °C for 1 hour. Figure 8 shows the optical image of the integrated ammonia sensor after the post-process.

## 5. Results and Discussion

**Figure 9.**
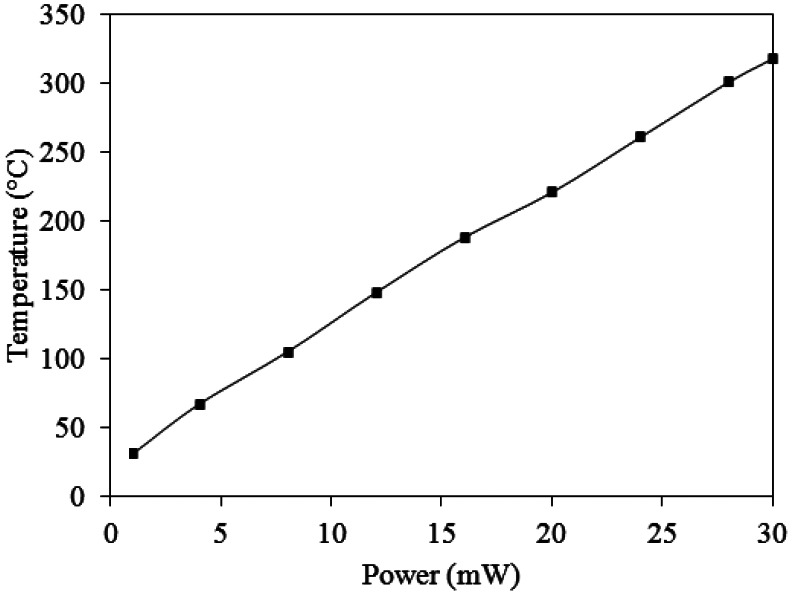
Measured results of the working temperature generated by the heater.

The working temperature of the ammonia sensor was provided by the heater. In order to characterize the performance of the heater, an infrared thermometer and a power supply were used to measure the working temperature. The power supply provided a power to the heater, and the infrared thermometer detected the working temperature of the ammonia sensor. Figure 9 shows the measured results of the working temperature generated by the heater. According to the results in Figure 9, the working temperature generated by the heater is given by:

(3) T=9.8P+26.5

where *P* represents the input power of the heater. In accordance with Equation (3), the heater can produce the working temperature of 280 °C when supplying a power of 25.9 mW to the heater.

**Figure 10.**
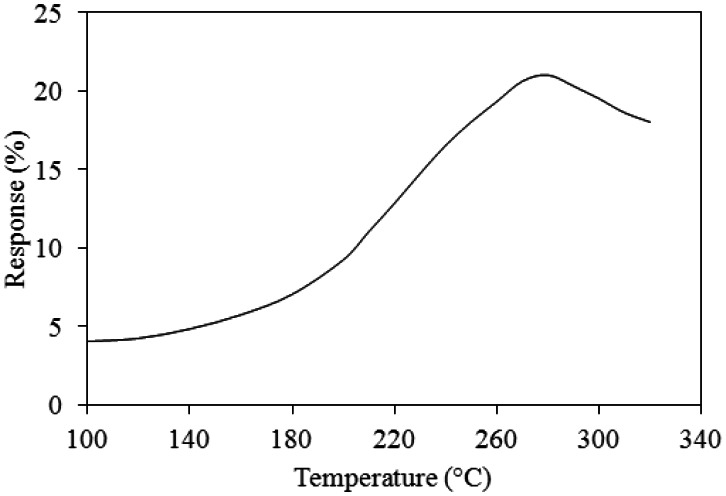
Relation between response and working temperature for the ammonia sensor at 50 ppm NH_3_.

A test chamber, an oscilloscope, a power supply and a LCR meter (LCR-819, Good Will Instrument Co. Taipei, Taiwan) were employed to test the performances of the ammonia sensor chip. The test chamber included a control valve, a calibration ammonia sensor and a pump. The control valve was utilized to tune ammonia input source. The calibration ammonia sensor monitored ammonia concentration in the test chamber. The pump was used to exhaust ammonia gas from the test chamber upon completion of the test. In order to obtain the best working temperature of the ammonia sensor, the sensor without the readout circuit was measured. The ammonia sensor chip without the readout circuit was set in the test chamber. The control valve tuned ammonia source to enter the test chamber. When the calibration ammonia sensor displayed the concentration of 50 ppm, the control valve was closed, so that the concentration of the test chamber kept at constant. Then, the power supply provided power to the heater. The heater supplied different working temperatures to the ammonia sensor, and the LCR meter recorded the resistance variation of the sensor. Figure 10 shows the relation between response and working temperature for the ammonia sensor at 50 ppm NH_3_. The response is defined as $\left|\frac{R_{s}-R_{0}}{R_{0}}\right|\times 100\%$, where *R_s_* is the resistance variation of the sensor sensing ammonia and *R_o_* is the initial resistance of the sensor. The results depicted that the best working temperature for the sensor was 280 °C. Finally, ammonia gas in the test chamber was exhausted by the pump when the test was finished.

**Figure 11.**
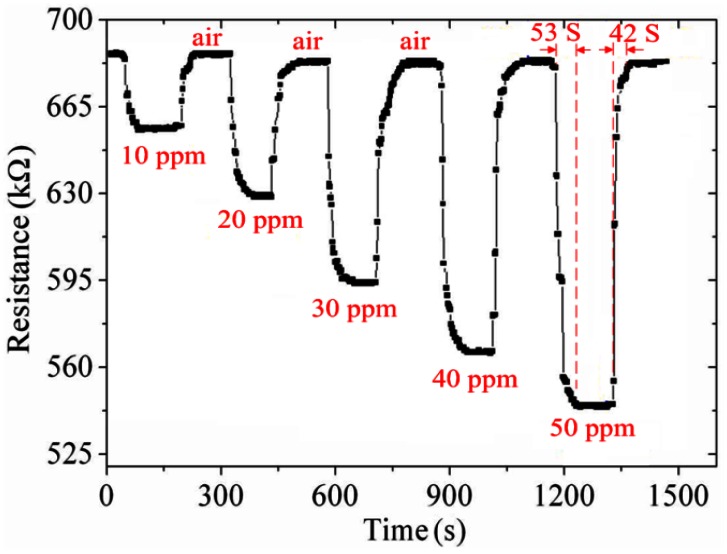
Test of the ammonia sensor.

As shown in Figure 10, the best working temperature of the ammonia sensor was 280 °C. The ammonia sensor chip without the readout circuit was set in the test chamber, and the heater provided a working temperature of 280 °C. The LCR meter was used to measure the resistance variation under different ammonia concentrations. Figure 11 shows the resistance variation of the ammonia sensor at different ammonia concentrations. The measured results showed that the initial resistance of the ammonia sensor was 680 kΩ in air, and the resistance of the sensor decreased to 545 kΩ at 50 ppm NH_3_.

**Figure 12.**
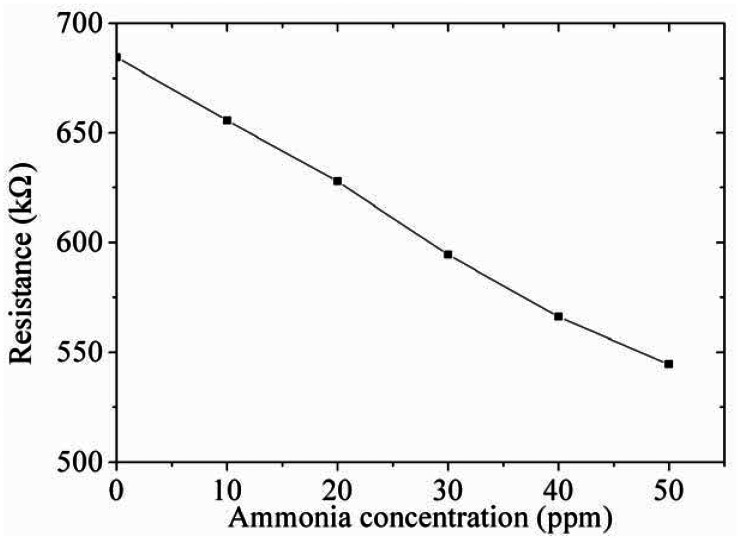
Measured results of the resistance for the ammonia sensors.

As shown in Figure 11, the ammonia sensor had a response time of 52 ± 1 s at 50 ppm NH_3_ and a recovery time of 41 ± 1 s at 50 ppm NH_3_. Figure 12 presents the relation between the resistance and ammonia concentration for the ammonia sensor. As the concentration of ammonia increased, the resistance of the ammonia sensor decreased.

**Figure 13.**
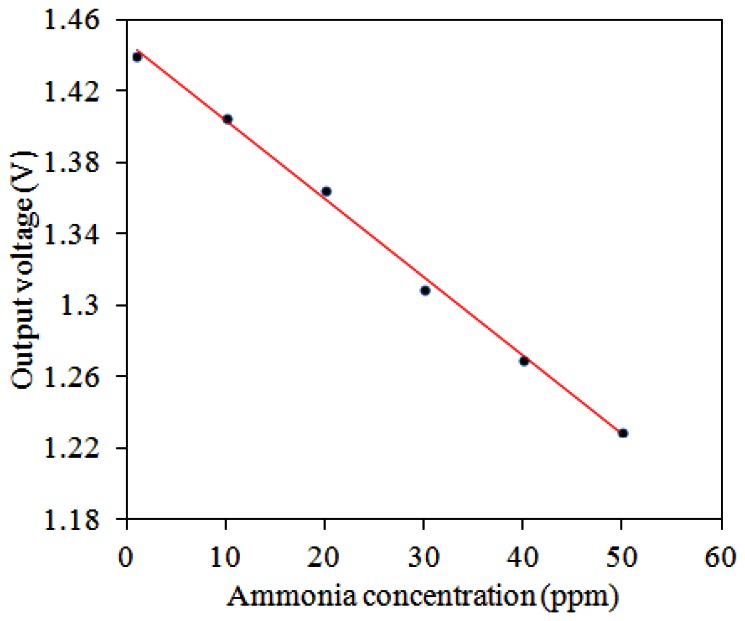
Measured results of the output voltage for the ammonia sensors.

The ammonia sensor with readout circuit was set in the test chamber, and the heater supplied a working temperature of 280 °C to the sensitive film. A bias voltage of 3.3 V and the input voltages V_in1_ = 0.6 V and V_in2_ = 0.9 V were provided to the readout circuit by the power supply. The ammonia sensor with readout circuit was tested under different ammonia concentrations, and the oscilloscope was used to record the output voltage of the ammonia sensor. Figure 13 shows the relation between the output voltage and ammonia concentration for the ammonia sensor. In this measurement, ammonia gas was supplied from 1 to 50 ppm. As the concentration of ammonia gas changed from 1 to 50 ppm, the output voltage of the ammonia sensor decreased from 1.44 to 1.23 V. The variation of the output voltage was 210 mV in 1–50 ppm NH_3_. Hence, the sensitivity of the integrated ammonia sensor was 4.1 mV/ppm.

An ammonia sensor integrated with a readout circuit, proposed by Yang *et al.* [15], was manufactured by the commercial 0.35 µm CMOS process and a post-process. The sensitive material of the ammonia sensor was zinc oxide, and its sensitivity was 1.5 mV/ppm. Liu *et al.* [16] also used the commercial CMOS process to fabricate an ammonia sensor integrated with a sensing circuit. The sensitive material of the sensor was polyaniline, and the sensor had a sensitivity of 0.88 mV/ppm. A comparison to Yang *et al.* [15] and Liu *et al.* [16], the sensitivity of the sensor in this work exceeds that of Yang *et al.* [15] and Liu *et al.* [16].

## 6. Conclusions

An ammonia sensor integrated with a readout circuit and a heater was successfully fabricated using the commercial 0.18 μm CMOS process and a post-process. The post-processing did not affect the function of the readout circuit, so it was compatible with the commercial CMOS process. The ammonia sensor contained a sensitive film and the interdigitated electrodes. The sensitive film of the ammonia sensor was zirconium dioxide prepared by the sol-gel method, and the film was deposited on the interdigitated electrodes. The heater was located under the interdigitated electrodes, and it provided a working temperature of 280 °C to the sensitive film. The resistance of the sensor changed as the sensitive film adsorbed or desorbed ammonia gas. The readout circuit converted the resistance variation of the sensor into the output voltage. The experimental results showed that the resistance of the sensor decreased from 680 to 545 kΩ as the ammonia gas concentration changed from 1 to 50 ppm at 280 °C. The sensitivity of the ammonia sensor was 4.1 mV/ppm. The response and recovery times were 52 ± 1 s at 50 ppm NH_3_ and 41 ± 1 s at 50 ppm NH_3_, respectively.
